# Daily and Seasonal Rhythms in Immune Responses of Splenocytes in the Freshwater Snake, *Natrix piscator*

**DOI:** 10.1371/journal.pone.0116588

**Published:** 2015-02-27

**Authors:** Manish Kumar Tripathi, Ramesh Singh, Atanu Kumar Pati

**Affiliations:** 1 Department of Zoology, Udai Pratap Autonomous College, Varanasi 221 002, India; 2 School of Life Sciences, Pt. Ravishankar Shukla University, Raipur 492 010, India; Pennsylvania State University, UNITED STATES

## Abstract

Present study was designed to examine daily and seasonal variability in the innate immune responses of splenocytes in the fresh water snake, *Natrix piscator*. Animals were mildly anesthetized and spleen was aseptically isolated and processed for macrophage phagocytosis, NBT reduction, nitrite production, splenocyte proliferation and serum lysozyme activity. Samples were collected at seven time points, viz., 0000, 0400, 0800, 1200, 1600, 2000 and 0000 h during three different seasons, namely summer, winter and spring. Cosinor analysis revealed that percent phagocytosis had a significant 24-h rhythm during summer and spring seasons. The peaks of rhythms in NBT reduction and nitrite release occurred in the morning hours at 10.88 h and 8.31 h, respectively, in winter. A significant 24-h rhythm was also observed in lysozyme concentration and splenocyte proliferation (both Basal and Concanavalin A stimulated) in all three seasons. A significant phase shift in splenocyte proliferation was obtained with a trend of delayed phase shift from winter to spring and from spring to summer. Of the nine variables, significant annual (seasonal) rhythms were detected in almost all variables, excluding phagocytic and splenosomatic indices. All rhythmic variables, except spleen cellularity, exhibited tightly synchronized peaks coinciding with the progressive and recrudescence phases of annual reproductive cycle. It is concluded that the snake synchronizes its daily and seasonal immune activity with the corresponding external time cues. The enhancement of immune function coinciding with one of its crucial reproductive phases might be helping it to cope with the seasonal stressors, including abundance of pathogens, which would otherwise jeopardize the successful reproduction and eventual survival of the species.

## Introduction

Many behavioral and physiological functions exhibit rhythms with varying frequencies. Most of these rhythms are endogenous [1] and retain abilities to synchronize with the day-night cycles or with other natural periodic cues. The daily rhythms provide a temporal framework necessary for maintaining homeostasis. However, all daily rhythms may not be circadian rhythms (CRs). The CR exhibits 24-h period, when synchronized with the day-night cycle of the nature, but it free runs and shows a period slightly longer or shorter than 24 h, when the organism showing CR is kept under constant conditions of light, temperature and other known periodic signals. Circadian rhythm enables organisms to maintain temporal harmony among numerous molecular, biochemical, physiological and behavioral processes vis-à-vis changes in the environmental factors.

The circulating formed elements in the peripheral blood, one of the important components of the immune system, show highly reproducible circadian rhythms. The circadian variations in number of circulating formed elements in the peripheral blood may not only largely be due to changes in the distribution, but may also relate to circadian changes in cell production and release and/or destruction. The reactivity of circulating blood cells also varies predictably as a function of time, as circadian rhythm has been reported in responsiveness of human and murine lymphocytes *in vitro* to mitogens [2]. The bone marrow represents an actively proliferating tissue with rapid turnover; and cell proliferation in bone marrow is not constant, but undergoes rhythmic variations [3–5]. Based on number of scholarly studies, a rhythmic immune network has been shown to operate in mammals long ago [6].

Phagocytic activity of neutrophils/heterophils of blood, the macrophages of spleen and peritoneum also exhibit circadian rhythms in birds and mammals. Similar daily rhythms in chemotaxis and neutrophil migration have been reported in many original reports [7–9]. Most research in this field is focused on the molecular and genetic aspects, with extensive work on *Drosophila* and mouse; however, the study of alternative animal models still represent a useful approach to understanding how the vertebrate circadian system is organized, and how this time-keeping system has changed during the course of vertebrate evolution. Attempts to understand mammalian immune system will be aided by a more systematic approach to investigating immune function across vertebrate groups. Reptiles are the crucial phylogenic group that gave rise to the evolution of both birds and mammals. Reptiles are the only ectothermic amniotes, and therefore, become a pivotal group for the study in order to provide significant insights into both the evolution and functioning of the rhythmicity in immune system. Reptiles have an extended terrestrial lifestyle and they show direct development without metamorphosis unlike the amphibians. Reptiles are generally long-lived, with an extended period of growth and maturation early in life. However, reptiles are unable to regulate their internal body temperature, and undergo strong seasonal changes in behavior associated with environmental temperatures. Collectively, these characteristics may have profound effects on how reptiles partition energy resources to self-maintenance activities. In reptiles that occupy a key position in the phylogeny of vertebrate evolution, chronobiological study on immune cells and its reactivity is inadequate. Although circadian rhythm in eosinophil has been reported in a lizard species [10] and in this snake species [11–13], no substantial information is available on the possibilities of existence of multi-frequency rhythms in immune system of reptiles. The present study was undertaken to throw light on daily and seasonal variations in immune responses in reptiles, using *Natrix piscator* as an ophidian model.

## Material and Methods

### Animals

Live freshwater snakes (> 120 g) were procured from a local supplier, who caught these animals in the suburbs of Varanasi (28^0^ 18’ N; 83^0^ 1’ E). In this study, we used male snakes only, especially because gender dependent innate immune responses have been reported in reptiles [14, 15]. Animals were housed in vivarium (wood and wire net cages; size 50x30x30 cm). Each cage had wooden floor and frame with three sides fitted with wire mesh and one side with a flap door and a small window. Each cage had an earthen bowl (4 L capacity) filled with water to accommodate 4–5 snakes. Snakes were fed small fishes once a week. Cages were cleaned and water in the bowl replenished the next day following feeding. Animals were acclimated to the laboratory conditions for two weeks; thereafter, experiments were performed. This study was approved by the Institutional Ethics Committee, Udai Pratap Autonomous College, Varanasi, India. The guidelines of the Institutional Ethics Committee and the committee for the purpose of control and supervision of experiment on animals (CPCSEA), the Ministry of Statistics and Program Implementation, Government of India, were strictly followed.

### Chemicals

Tetrazolium dye, NBT (Nitroblue Tetrazolium) and MTT [3-(4, 5-dimethylthiozol-2-yl)-2, 5 diphenyl tetrazolium bromide]; mitogens [concanavalin A (Con A), phytohemagglutinin (PHA) and lipopolysaccharide (LPS)]; and melatonin were purchased from Sigma Chemicals. Culture medium (RPMI-1640), lymphocyte separation medium (HiSep), L-glutamine, gentamycin, fetal bovine serum (FBS), dimethyl sulfoxide (DMSO), and other chemicals were purchased from Himedia Laboratories Pvt. Ltd. (India). The culture medium was supplemented with 1 μl ml^-1^ gentamycin, 10 μl ml^-1^ of 200 mM L-glutamine, 10 μl ml^-1^ anti-anti (Gibco) and 5% FBS and referred to as complete culture medium.

### Experiment

This study was designed to understand daily and seasonal variabilities in the immune response of splenocytes in the fresh water snake, *Natrix piscator*. This study was performed in three seasons, *viz*. summer (May), winter (December) and spring (March). In each season, animals were divided into seven groups as outlined below: The animals of Group-1 to Group-7 were sacrificed, respectively, at 0400, 1200, 2000, 0000, 0800, 1600, and 0000 h.

Each group contained 5 animals. The animals of the Groups 1 to 3 were anesthetized mildly at the designated time points and blood was sampled through cardiocentesis in heparinized tubes and was centrifuged to obtain plasma. Plasma was kept at—20°C for a week and then utilized for analysis of lysozyme concentration. Spleen was excised aseptically, weighed, kept in cool (4°C) culture medium separately and processed immediately to study the immune parameters. Following two days, groups 4 to 7 animals were anesthetized mildly at the given time points, and blood and spleen were sampled as described above.

### Preparation of macrophage monolayer

Splenic macrophage monolayer was prepared, and phagocytic assay was performed following the method of Mondal and Rai [14,16]. Briefly, excised spleen was macerated individually through a nylon strainer of pore size <100 μm into complete culture medium (2 ml per spleen) to get single cell suspension under a sterile laminar flow hood. Cell viability was checked through trypan blue exclusion test, which exceeded 95%, and spleen cellularity (number of cells mg^–1^ tissue) was determined with help of hemocytometer.

Splenic cell suspension (200 μl) was flooded onto individual pre-washed and sterilized glass slides. Phagocytic macrophages were allowed to adhere by incubating the slides at 25°C in humidified CO_2_ atmosphere for 90 min. Non adherent cells were washed off with 0.2 M phosphate buffer saline (PBS; pH 7.2). The splenic macrophage monolayer was prepared in duplicate from each spleen. In the adherent cell population, more than 90% of the cells were macrophages as judged by their morphology.

### Phagocytic assay

For phagocytic assay, the yeast cells were used as target cell. The yeast cell suspension was prepared by mixing 20 mg of commercial baker’s yeast (*Saccharomyces cerevisae*) in 10 ml of 0.2 M PBS. The suspension was kept at 80°C for 15 min. The cell suspension was washed three times in PBS and finally suspended in complete culture medium to get a concentration of 1×10^8^ cells ml^-1^.

The prepared macrophage monolayer, as above, was flooded with yeast cell suspension, and phagocytosis was allowed to proceed by incubating at 25°C in humidified CO_2_ atmosphere for 90 min. Then, slides were rinsed three times in PBS, fixed in methanol, stained with Giemsa, and examined under oil immersion. For each slide, a total of 100 macrophages were examined randomly without any predetermined sequence. The phagocytic index was determined by calculating the average number of yeast cells engulfed by single macrophage. The percent phagocytosis was calculated by dividing the number of macrophages showing phagocytosis by 100.

### NBT assay

Superoxide anion production by phagocyte was determined as the reduction of NBT. NBT is a water soluble, yellow colored and membrane permeable dye. It is reduced into NBT-diformazan (Purple color) by superoxide. NBT assay was performed following the methods of Berger and Slapnickova [9]. Spleen cells were counted and adjusted to 2×10^6^ cells ml^–1^ in complete RPMI. Fifty microlitres of cell suspension (1×10^5^ cells) was mixed with 50 μl of RPMI containing NBT (1 mg ml^–1^) in culture plate (96 well) in triplicates from each spleen. Wells with culture medium (100 ml) without cells in triplicates served as blank. Plate was then incubated in CO_2_ atmosphere at 25°C for 2 h, centrifuged at 700xg, washed with PBS and fixed in 70% methanol. Twenty microlitres of 0.1% triton X-100 was mixed in each well. The formazan crystals, present inside the cells, were dissolved by mixing 120 μl KOH (2 M) and 140 μl DMSO in each well. Optical density was measured at 620 nm with the help of ELISA plate reader (Thermo Multiscan). Following blank subtraction, triplicates were averaged.

### Nitrite assay

Nitric oxide (NO) is a major effector molecule of macrophage cytotoxicity. It is a highly unstable compound produced from L-arginine by enzyme Nitric oxide synthase (NOS). Soon after production, NO decomposes to other nitrogen oxides such as nitrate (NO_3_^–^) and nitrite (NO_2_^–^) popularly known as Reactive Nitrogen Intermediate (RNI) [17]. So, nitrite was assayed as a marker of cytotoxicity. Nitrite content was measured by the method of Ding et al. [18]. Briefly, 100 μl of splenocytes (1×10^6^ cells ml^–1^) was added to each well of 96 well culture plate. After two hours of incubation at 25°C, cells were washed with PBS. Fresh culture medium (100 μl) was added to each well and plates were incubated in CO_2_ atmosphere at 25°C for 24 h, then centrifuged at 200xg and supernatant was collected. Equal volume of supernatant and Griess reagent (1% sulfanilamide in 3 N HCl and 0.1% naphthylenediamine dihydrochloride in distilled water) are mixed and optical density of the solution was measured at 540 nm with the help of ELISA plate reader (Thermo Multiscan). Culture medium alone without any cell served as blank. All the samples were taken in triplicate. Following blank subtraction, triplicates were averaged.

### Splenic lymphocyte proliferation assay

The lymphocyte proliferation was assessed using colorimetric assay based on tetrazolium salt (MTT) following the methods of Berridge et al. [19]. The colorimetric method, utilizing tetrazolium salts, has been an advantageous alternative method measuring lymphoproliferation [20]. In metabolically active cells, tetrazolium salts are incorporated into active mitochondria, the tetrazolium rings of MTT is cleaved by mitochondrial dehydrogenase enzyme and bioreduced into dark blue formazan crystals which are impermeable to the cell membrane. Solubilisation of cells by the addition of a detergent results in liberation of the crystals. The quantity of formazan product as measured by amount of absorbance 570 nm is directly proportional to the number of living cells in culture [21]. Thus, quantifying the conversion of salts by mitochondrial dehydrogenases in blue colored formazan product provides a measure cell number during last hours of *in vitro* culture. The accumulation of colored formazan products is positively correlated with incorporation of ^3^H-thymidine into cellular DNA in the S-Phase of cell division during last hours of *in vitro* culture, which is a direct measure of blastogenesis under the conditions of mitogenic stimulation [22].

Splenic single cell suspension prepared as above was treated with hemolysate buffer (0.15 M NH_4_Cl, 10 mM KHCO_3_, 0.1 mM Na_2_EDTA, pH 7.2), washed with 0.2 M PBS (pH 7.2) twice and resuspended in complete culture medium. Splenic lymphocytes were isolated by density gradient centrifugation using HiSep (Density 1.077 g ml^-1^). The cell suspension was overlaid on equal volume of HiSep and centrifuged at 400xg for 30 min with brakes off at 8°C. Following centrifugation, lymphocyte fraction at the interface between medium and HiSep was carefully aspirated, washed twice with PBS, counted and assessed for viability on a hemocytometer through trypan blue exclusion test. Viable cells (>95%) were adjusted at 2×10^6^ cells ml^-1^ in complete culture medium.

Basal as well as mitogen (Con A) induced *in vitro* lymphocyte proliferation was assessed. Stock solution of mitogen was made in 0.2 M PBS (pH 7.2) at a concentration of 1 mg ml^-1^, and further dilution was made with culture medium to get a concentration of 10 μg ml^-1^. Flat bottom 96 well culture plates were used. To study spontaneous or basal proliferation, 50 μl cell suspension was seeded into well of culture plate along with 50 μl of mitogen-free culture medium, while to study induced one, 50 μl of mitogen and 50 μl of cell suspension were seeded. Additional well containing only 100 μl of culture medium served as blank. All assays were made in triplicates form each experimental animal. Plates were incubated in humidified CO_2_ atmosphere at 25°C for 48 h, after which 10 μl of MTT reagent (5 mg ml^-1^) was added to each well, and plates were again incubated overnight in humidified CO_2_ atmosphere at 25°C. Following incubation, the supernatant was aspirated, and 100 μl of DMSO was added to each well to solubilize the formazan crystals. Absorbance was measured at 570 nm with the help of ELISA plate reader (Thermo Multiscan). Following blank subtraction, triplicates were averaged.

### Lysozyme assay

Lysozyme activity was measured using a standard turbidity assay, as previously described by Demers and Bayne [23]. Hen egg lysozyme (HEL; Sigma, St. Louis, MO) was used as standard. Stock solution (1 mg ml^-1^) of standard HEL was prepared in 0.1 M phosphate buffer (pH 5.9) and was serially diluted in phosphate buffer to produce a standard curve of 40, 20, 10, 5, 2.5, 1.25, 0.6, 0.3, and 0 μg ml^-1^. Aliquots (25 μl per well) of each concentration of standard as well as test plasma sample were added to a 96-well plate in triplicate. The solution of *Micrococcus lysodeikticus* (175 μl per well) was quickly added to each of the sample and standard wells. The solution of *Micrococcus lysodeikticus* was prepared by dissolving 20 mg lyophilized bacterial powder (Sigma, St. Louis, MO) into 100 ml phosphate buffer. Triplicate well containing plasma received 175 μl phosphate buffer and served as blank. Absorbance was measured at 450 nm with ELISA plate reader (Thermo Multiscan) immediately (T_0_) and again after five minutes (T_5_). Absorbance unit (AU) value for the blank sample well was subtracted from the average of the triplicate sample wells to compensate for any hemolysis in the samples. AU values at T_5_ were subtracted from AU values at T_0_ to determine the change in absorbance ∆_AU_5. The resultant ∆_AU_5 value was converted to HEL concentration (μg μl^-1^) via linear regression of the standard curve.

### Statistical analysis

Data are presented as mean ± 1 SEM as function of time and season. The data were subjected to Cosinor rhythmometry [24,25] for validation of diurnal and annual rhythms. The rhythms were characterized by three parameters, such as Mesor (M, rhythm-adjusted mean; it equals the arithmetic mean, when data were sampled at equidistant time intervals), the amplitude (A, half of the difference between minimum and maximum in the biological function), and the acrophase or peak (∅, time of maximum in the fitted cosine function, with local midnight for diurnal rhythm and local midnight of December 22 for annual rhythm, as ∅ reference). Data were also presented separately as function of seasons, namely summer (May), winter (December) and spring (March) for further examination of seasonal variability. One-way and two-way analyses of variance (ANOVAs) were used to validate time effects both along the diurnal and annual (seasonal) time scales, respectively. All statistical analyses, except Cosinor rhythmometry, were performed using SPSS for Windows. All null hypotheses were tested at p ≤ 0.05.

## Result and Discussion

**Fig 1:**
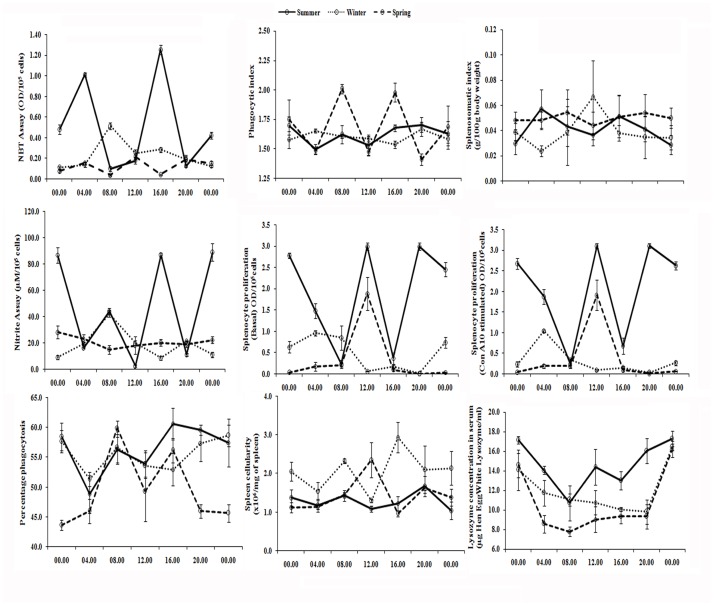
Variations in different immune parameters in the freshwater snake, *Natrix piscator* during 24-h period.

**Fig 2:**
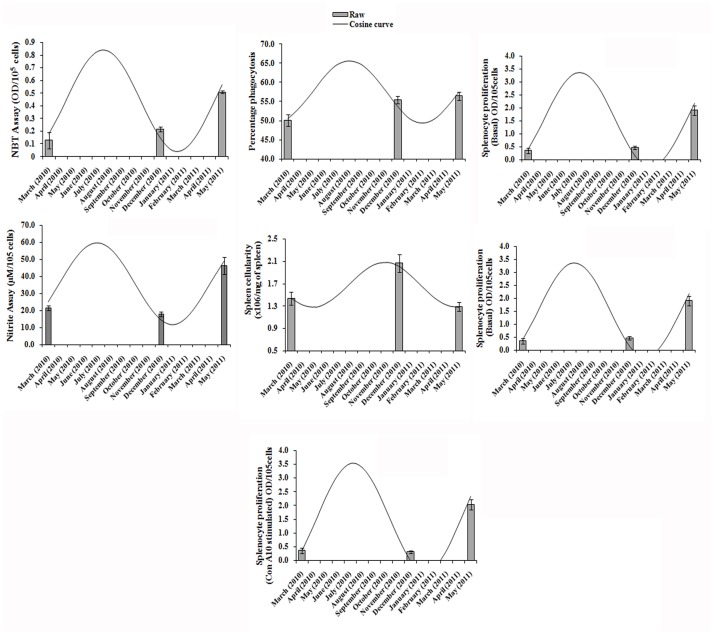
Circannual rhythm in different immune parameters in the freshwater snake, *Natrix piscator*.

**Fig 3:**
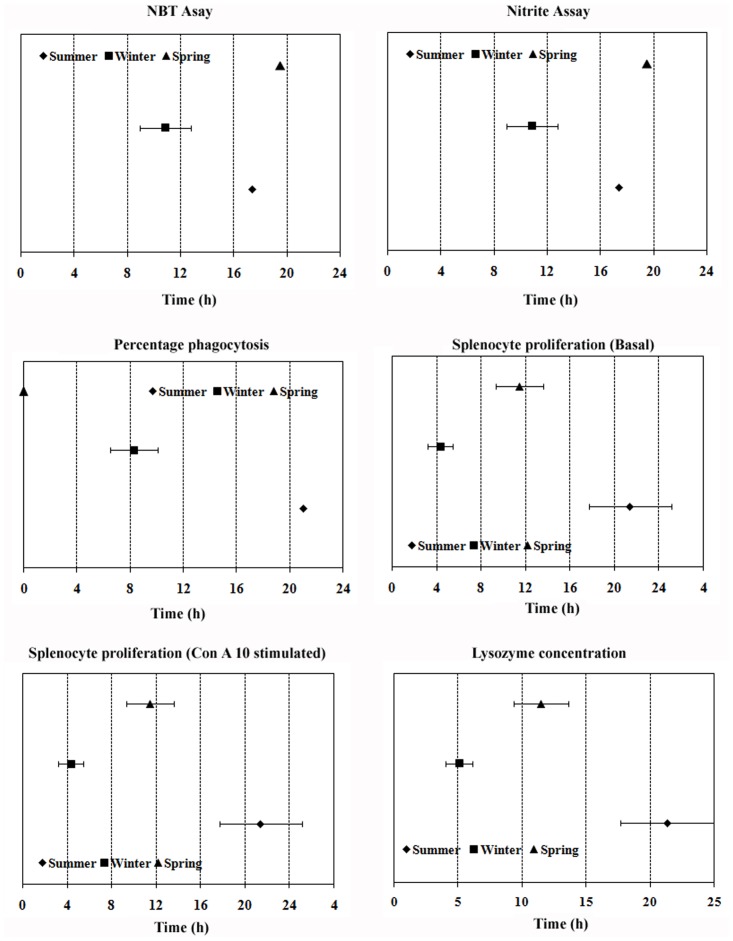
Peak map showing 24-h rhythm in different immune parameters in the freshwater snake, *Natrix piscator*.

**Fig 4:**
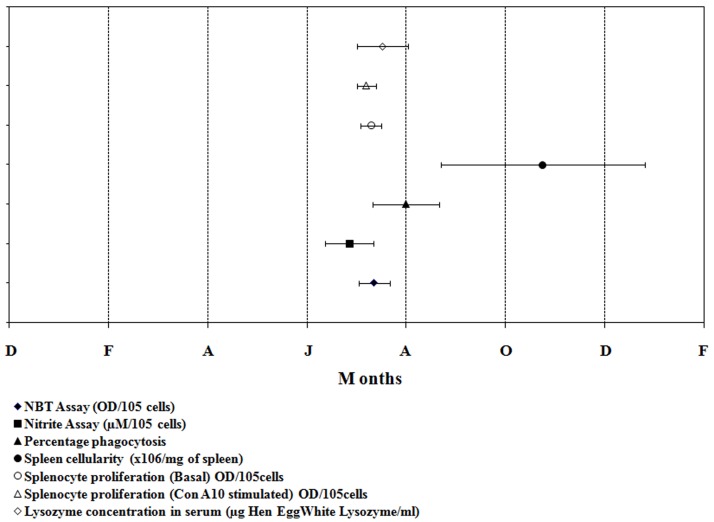
Annual peak map showing peaks in different immune parameters in the freshwater snake, *Natrix piscator*.

Variability observed in different immune parameters along 24-h and annual (seasonal) time scales are graphically presented in Figs. 1 and 2, respectively. The results emanated from Cosinor rhythmometry are summarized in Tables 1, 2, 3 for daily rhythms and in Table 4 for seasonal rhythms. The results from 2-way ANOVA are illustrated in Table 5. Characteristics of daily variability in immune responses of splenocytes are presented in Tables 6, 7, 8. The diurnal peak maps were constructed only for those parameters in which at least one significant rhythm appeared, irrespective of season (Fig. 3A, B) and annual peak map was constructed for seven variables (Fig. 4).

**Table 1:** Cosinor and ANOVA summaries: Characteristics of daily variability in immune responses of splenocytes in summer.

Variable	Data point	Rhythm detection (*p*)	Rhythm adjusted mean (±SE)	Amplitude (A) (95% CL)	Acrophase (Ø in h) (95% CL)	Time effect ANOVA F [df]; p value
NBT Reduction	60	<**0.001**	0.24 ± 0.02	0.12 (0.07, 0.17)	10.88 (8.95, 12.81)	20.001 [6, 53]; <**0.001**
Nitrite Release	60	<**0.001**	19.11 ± 1.19	9.44 (4.98, 13.9)	08.31 (6.51, 10.11)	40.593 [6, 53]; <**0.001**
Phagocytosis	25	0.38	55.13 ± 1.05	2.04	22.98	1.058 [6, 18]; 0.422
Phagocytic index	25	0.62	1.60 ± 0.02	0.03	01.61	1.165 [6, 18]; 0.367
Splenosomatic index	28	0.43	0.04 ± 0.01	0.01	13.07	0.596 [6, 21]; 0.73
Spleen cellularity	21	0.39	2.06 ± 0.16	0.34	18.07	2.313 [6, 14]; 0.092
Basal Splenocyte proliferation	60	<**0.001**	0.46 ± 0.04	0.51 (0.35, 0.67)	04.35 (3.22, 5.48)	13.427 [6, 53]; <**0.001**
Con A stimulated Splenocyte proliferation	60	<**0.001**	0.31 ± 0.03	0.38 (0.26, 0.50)	05.08 (4.01, 6.15)	66.921 [6, 53]; <**0.001**
Plasma Lysozyme	27	**0.02**	11.70 ± 0.54	2.25(0.32, 3.74)	01.49 (21.85, 5.13)	3.985 [6, 20]; **0.009**

**Table 2:** Cosinor and ANOVA summaries: Characteristics of daily variability in immune responses of splenocytes in winter.

Variable	Data point	Rhythm detection (*p*)	Rhythm adjusted mean, (±SE)	Amplitude (A) (95% CL)	Acrophase (Ø in h) (95% CL)	Time effect ANOVA F [df]; p value
NBT Reduction	60	<**0.001**	0.24 ± 0.02	0.12 (0.07, 0.17)	10.88 (8.95, 12.81)	20.001 [6, 53]; <**0.001**
Nitrite Release	60	<**0.001**	19.11 ± 1.19	9.44 (4.98, 13.9)	08.31 (6.51, 10.11)	40.593 [6, 53]; <**0.001**
Phagocytosis	25	0.38	55.13 ± 1.05	2.04	22.98	1.058 [6, 18]; 0.422
Phagocytic index	25	0.62	1.60 ± 0.02	0.03	01.61	1.165 [6, 18]; 0.367
Splenosomatic index	28	0.43	0.04 ± 0.01	0.01	13.07	0.596 [6, 21]; 0.73
Spleen cellularity	21	0.39	2.06 ± 0.16	0.34	18.07	2.313 [6, 14]; 0.092
Basal Splenocyte proliferation	60	<**0.001**	0.46 ± 0.04	0.51 (0.35, 0.67)	04.35 (3.22, 5.48)	13.427 [6, 53]; <**0.001**
Con A stimulated Splenocyte proliferation	60	<**0.001**	0.31 ± 0.03	0.38 (0.26, 0.50)	05.08 (4.01, 6.15)	66.921 [6, 53]; <**0.001**
Plasma Lysizyme	27	**0.02**	11.70 ± 0.54	2.25 (0.32, 3.74)	01.49 (21.85, 5.13)	3.985 [6, 20]; **0.009**

**Table 3:** Cosinor and ANOVA summaries: Characteristics of daily variability in immune responses of splenocytes in spring.

Variable	Data point	Rhythm detection (*p*)	Rhythm adjusted mean, (±SE)	Amplitude (A) (95% CL)	Acrophase (Ø in h) (95% CL)	Time effect ANOVA F [df]; p value
NBT Reduction	63	0.69	0.13 ± 0.01	0.014	19.5	17.979 [6, 56]; <**0.001**
Nitrite Release	57	0.16	20.55 ± 1.48	3.795	0.005	1.222 [6, 50]; 0.311
Phagocytosis	23	**0.005**	50.69 ± 1.19	5.97 (1.67, 10.27)	11.67 (8.29, 15.05)	6.927 [6, 16]; **0.001**
Phagocytic index	23	0.52	1.72 ± 0.06	0.09	12.41	6.17 [6, 16]; **0.002**
Splenosomatic index	28	0.99	0.05 ± 0.004	0.001	20.79	0.091 [6, 21]; 0.997
Spleen cellularity	21	0.18	1.48 ± 0.11	0.29	12.00	5.201 [6, 14]; **0.005**
Basal Splenocyte proliferation	63	<**0.001**	0.43 ± 0.08	0.58 (0.30, 0.86)	11.47 (9.34, 13.60)	19.302 [6, 56]; <**0.001**
Con A stimulated Splenocyte proliferation	63	<**0.001**	0.44 ± 0.08	0.57 (0.30, 0.84)	11.47 (9.36, 13.58)	23.908 [6, 56]; <**0.001**
Plasma Lysizyme	27	**0.001**	10.38 ± 0.55	3.06 (1.19, 4.93)	23.41 (20.57, 2.25)	10.675 [6, 20]; <**0.001**

**Table 4:** Cosinor summary: Characteristics of seasonal variability in immune responses of splenocytes.

Variable	Data points (N)	Rhythm detection (P)	Mean (M±SE)	Amplitude	Acrophase (month)	Acrophase (date)
NBT Assay (OD/10^5^ cells)	174	<0.001	0.44 ± 0.03	0.40 (0.26, 0.54)	7.35 (7.04, 7.66)	August 02 (July 23, August 11)
Nitrite Assay (μM/10^5^ cells)	171	<0.001	35.49 ± 2.81	23.92 (12.70, 35.14)	6.85 (6.36, 7.34)	July 18 (July 03, August 01)
Percentage phagocytosis	84	0.003	57.54 ± 1.13	8.08 (2.45, 13.71)	7.99 (7.32, 8.66)	August 21 (August 01, September 10)
Phagocytic index	84	0.07	1.58 ± 0.03	0.15	2.14	February 25
Splenosomatic index (g/100g body weight)	84	0.31	0.04 ± 0.01	0.01	2.23	February 28
Spleen cellularity (x 10^6^/mg of spleen)	63	<0.001	1.68 ± 0.12	0.40 (0.19, 0.61)	10.75 (8.70, 12.80)	November 13 (September 12, February 14)
Splenocyte proliferation (Basal OD/10^5^ cells)	165	<0.001	1.54 ± 0.11	1.83 (1.35, 2.31)	7.29 (7.08, 7.50)	July 31 (July 25, August 06)
Splenocyte proliferation (Con A 10 stimulated OD/10^5^ cells)	164	<0.001	1.55 ± 0.10	1.98 (1.53, 2.43)	7.19 (7.00, 7.38)	July 28 (July 22, August 03)
Lysozyme concentration in serum (μg Hen Egg White Lysozyme/ml)	81	<0.001	14.14 ± 0.62	4.28 (3.66, 4.90)	7.52 (7.01, 8.03)	August 07 (July 22, August 22)

**Table 5:** Two-way ANOVA summary illustrating effects of season, time of the day and their interaction.

Variable	R^2^	Source	df	MS	F-value	P
NBT Assay (OD/10^5^ cells)	0.948	Season (S)	2	2.109	379.317	<0.001
		Time of the day (T)	6	0.472	84.896	<0.001
		S x T	12	0.705	126.827	<0.001
		Error	153	0.006	-	-
Nitrite Assay (μM/10^5^ cells)	0.908	Season (S)	2	14231.890	212.825	<0.001
		Time of the day (T)	6	3436.033	51.383	<0.001
		S x T	12	4728.039	70.704	<0.001
		Error	150	66.871	-	-
Phagocytosis (%)	0.546	Season (S)	2	351.368	12.735	<0.001
		Time of the day (T)	6	98.167	3.558	0.004
		S x T	12	67.836	2.459	0.011
		Error	63	27.590	-	-
Phagocytic index	0.556	Season (S)	2	0.043	2.056	0.136
		Time of the day (T)	6	0.084	4.036	0.002
		S x T	12	0.086	4.101	<0.001
		Error	63	0.021	-	-
Splenosomatic index (g/100g body weight)	0.151	Season (S)	2	0.001	1.138	0.327
		Time of the day (T)	6	0	0.245	0.96
		S x T	12	0	0.625	0.813
		Error	63	0.001	-	-
Spleen cellularity (x 10^6^/mg of spleen)	0.669	Season (S)	2	3.550	17.760	<0.001
		Time of the day (T)	6	0.275	1.376	0.246
		S x T	12	0.683	3.416	0.002
		Error	42	0.200	-	-
Splenocyte proliferation (Basal) OD/10^5^ cells	0.884	Season (S)	2	34.756	243.904	<0.001
		Time of the day (T)	6	5.142	36.085	<0.001
		S x T	12	5.088	35.709	<0.001
		Error	144	0.142	-	-
Splenocyte proliferation (Con A 10 stimulated OD/10^5^ cells)	0.922	Season	2	44.364	447.482	<0.001
		Time of the day (T)	6	5.456	55.030	<0.001
		S x T	12	4.326	43.631	<0.001
		Error	143	0.099	-	-
Lysozyme concentration in serum (μg Hen Egg White Lysozyme/ml)	0.703	Season	2	108.246	20.926	<0.001
		Time of the day (T)	6	73.842	14.275	<0.001
		S x T	12	6.770	1.309	0.238
		Error	60	5.173	-	-

**Table 6:** ANOVA summaries: Characteristics of daily variability in immune responses of splenocytes in summer.

Variable	00.00	04.00	08.00	12.00	16.00	20.00	00.00
NBT Assay (OD/10^5^ cells)	0.48 ± 0.05^c^	1.01 ± 0.02^b^	0.10 ± 0.02^d^	0.18 ± 0.03^d^	1.26 ± 0.05^a^	0.12 ± 0.01^d^	0.42 ± 0.03^c^
Nitrite release (μM/10^5^ cells)	86.61 ± 5.89^a^	16.41 ± 0.43^c^	44.30 ± 2.44^b^	2.58 ± 0.87^d^	86.99 ± 1.73^a^	11.33 ± 1.07^c^	88.98 ± 6.77^a^
Percentage phagocytosis	58.50 ± 2.33^a^	48.83 ± 3.16^b^	56.33 ± 2.16^ab^	54.00 ± 2.20^ab^	60.67 ± 2.67^a^	59.67 ± 0.84^a^	57.50 ± 4.03^a^
Phagocytic index	1.70 ± 0.08^a^	1.50 ± 0.04^a^	1.62 ± 0.08^a^	1.53 ± 0.08^a^	1.68 ± 0.03^a^	1.71 ± 0.06^a^	1.63 ± 0.10^a^
Splenosomatic index (g/100g body weight)	0.03 ± 0.01^a^	0.06 ± 0.02^a^	0.04 ± 0.02^a^	0.04 ± 0.01^a^	0.05 ± 0.02^a^	0.04 ± 0.01^a^	0.03 ± 0.01^a^
Spleen cellularity (x 10^6^/mg of spleen)	1.38 ± 0.22^a^	1.18 ± 0.17^a^	1.43 ± 0.13^a^	1.09 ± 0.08^a^	1.23 ± 0.18^a^	1.68 ± 0.26^a^	1.04 ± 0.23^a^
Splenocyte proliferation (Basal) OD/10^5^ cells	2.79 ± 0.06^ab^	1.49 ± 0.17^c^	0.23 ± 0.10^d^	3.00 ± 0.08^a^	0.37 ± 0.13^d^	3.00 ± 0.08^a^	2.46 ± 0.16^b^
Splenocyte proliferation (Con A 10 stimulated) OD/10^5^ cells	2.68 ± 0.12^b^	1.89 ± 0.18^c^	0.24 ± 0.10^c^	3.11 ± 0.05^a^	0.68 ± 0.20^d^	3.08 ± 0.08^a^	2.61 ± 0.12^b^
Lysozyme concentration in serum (μg Hen Egg White Lysozyme/ml)	17.13 ± 0.37^a^	14.07 ± 0.41^abc^	10.74 ± 1.79^c^	14.37 ± 1.81^abc^	12.99 ± 0.93^bc^	16.03 ± 1.26^ab^	17.27 ± 0.79^a^

Data are presented as Mean ± SE. Means having similar alphabets, as superscripts, are not statistically significant from each other at *p* < 0.05 (based on Duncan’s multiple-range test)

**Table 7:** ANOVA summaries: Characteristics of daily variability in immune responses of splenocytes in winter.

Variable	00.00	04.00	08.00	12.00	16.00	20.00	00.00
NBT Assay (OD/10^5^ cells)	0.11 ± 0.01^d^	0.14 ± 0.02^d^	0.52 ± 0.03^a^	0.25 ± 0.03^bc^	0.29 ± 0.02^b^	0.19 ± 0.04^cd^	0.13 ± 0.01^d^
Nitrite release (μM/10^5^ cells)	9.27 ± 1.62^c^	19.51 ± 1.18^b^	42.44 ± 2.65^a^	20.16 ± 1.86^b^	8.80 ± 1.52^c^	21.61 ± 0.44^b^	11.25 ± 2.02^c^
Percentage phagocytosis	57.67 ± 1.86^a^	51.33 ± 1.20^a^	56.67 ± 2.73^a^	53.67 ± 2.19^a^	53.00 ± 2.70^a^	57.40 ± 3.03^a^	58.67 ± 1.86^a^
Phagocytic index	1.58 ± 0.07^a^	1.65 ± 0.02^a^	1.61 ± 0.02^a^	1.59 ± 0.03^a^	1.54 ± 0.03^a^	1.67 ± 0.06^a^	1.58 ± 0.03^a^
Splenosomatic index (g/100g body weight)	0.04 ± 0.01^a^	0.02 ± 0.004^a^	0.04 ± 0.03^a^	0.07 ± 0.03^a^	0.04 ± 0.01^a^	0.04 ± 0.02^a^	0.04 ± 0.01^a^
Spleen cellularity (x 10^6^/mg of spleen)	2.06 ± 0.25^ab^	1.55 ± 0.24^b^	2.34 ± 0.06^ab^	1.31 ± 0.05^b^	2.94 ± 0.40^a^	2.11 ± 0.61^ab^	2.15 ± 0.44^ab^
Splenocyte proliferation (Basal) OD/10^5^ cells	0.64 ± 0.14^a^	0.96 ± 0.06^a^	0.85 ± 0.28^a^	0.07 ± 0.01^b^	0.17 ± 0.04^b^	0.01 ± 0.001^b^	0.74 ± 0.13^a^
Splenocyte proliferation (Con A 10 stimulated) OD/10^5^ cells	0.24 ± 0.06^bc^	1.05 ± 0.04^a^	0.34 ± 0.02^b^	0.10 ± 0.04^cd^	0.15 ± 0.04^cd^	0.04 ± 0.01^d^	0.28 ± 0.06^bc^
Lysozyme concentration in serum (μg Hen Egg White Lysozyme/ml)	14.09 ± 2.05^ab^	11.77 ± 1.31^bc^	11.07 ± 0.62^bc^	10.77 ± 1.22^bc^	10.07 ± 0.20^c^	9.83 ± 1.25^c^	16.44 ± 0.33^a^

Data are presented as Mean ± SE. Means having similar alphabets, as superscripts, are not statistically significant from each other at *p* < 0.05 (based on Duncan’s multiple-range test)

**Table 8:** ANOVA summaries: Characteristics of daily variability in immune responses of splenocytes in spring.

Variable	00.00	04.00	08.00	12.00	16.00	20.00	00.00
NBT Assay (OD/10^5^ cells)	0.08 ± 0.01^c^	0.16 ± 0.01^b^	0.04 ± 0.01^c^	0.22 ± 0.03^a^	0.05 ± 0.01^c^	0.20 ± 0.02^ab^	0.16 ± 0.02^b^
Nitrite release (μM/10^5^ cells)	28.42 ± 4.93^a^	23.20 ± 3.65^b^	15.18 ± 3.29^b^	18.34 ± 6.68^ab^	20.42 ± 2.47^ab^	19.10 ± 2.13^ab^	22.34 ± 2.60^ab^
Percentage phagocytosis	43.67 ± 0.88^c^	46.00 ± 2.08^c^	60.00 ± 1.15^a^	49.33 ± 5.04^bc^	56.20 ± 2.03^ab^	46.00 ± 1.15^c^	45.67 ± 1.45^c^
Phagocytic index	1.75 ± 0.17^ab^	1.49 ± 0.03^bc^	2.01 ± 0.04^a^	1.47 ± 0.03^bc^	1.98 ± 0.08^a^	1.41 ± 0.05^c^	1.68 ± 0.18^abc^
Splenosomatic index (g/100g body weight)	0.05 ± 0.01^a^	0.05 ± 0.01^a^	0.05 ± 0.02^a^	0.04 ± 0.01^a^	0.05 ± 0.02^a^	0.05 ± 0.01^a^	0.05 ± 0.01^a^
Spleen cellularity (x 10^6^/mg of spleen)	1.12 ± 0.13^b^	1.14 ± 0.06^b^	1.45 ± 0.07^b^	2.36 ± 0.45^a^	0.97 ± 0.08^b^	1.63 ± 0.06^b^	1.39 ± 0.22^b^
Splenocyte proliferation (Basal) OD/10^5^ cells	0.04 ± 0.01^b^	0.18 ± 0.11^b^	0.21 ± 0.04^b^	1.89 ± 0.39^a^	0.10 ± 0.03^b^	0.01 ± 0.002^b^	0.03 ± 0.01^b^
Splenocyte proliferation (Con A 10 stimulated) OD/10^5^ cells	0.05 ± 0.004^b^	0.20 ± 0.05^b^	0.19 ± 0.03^b^	1.91 ± 0.37^a^	0.10 ± 0.03^b^	0.02 ± 0.01^b^	0.07 ± 0.005^b^
Lysozyme concentration in serum (μg Hen Egg White Lysozyme/ml)	14.57 ± 1.22a	8.59 ± 0.90^b^	7.80 ± 0.50^b^	9.05 ± 1.29^b^	9.39 ± 0.76^b^	9.35 ± 1.25^b^	16.22 ± 0.82^a^

Data are presented as Mean ± SE. Means having similar alphabets, as superscripts, are not statistically significant from each other at *p* < 0.05 (based on Duncan’s multiple-range test)

Cosinor analysis revealed absence of statistically significant 24-h rhythm in fluctuation of splenosomatic index and spleen cellularity, irrespective of seasons. The percentage of phagocytosis exhibited significant 24-h rhythm in summer and spring seasons, but not in winter; and statistically significant 24-h rhythm in phagocytic index could not be validated for any one of the seasons. In percentage phagocytosis, phase shift was observed in spring as compared to summer season (Fig. 3A). The peak in percentage phagocytosis rhythm appeared in the evening at 17.83 h in summer; while the peak was recorded at noon (11.67 h) in spring season. Thus, the peak in the percentage of phagocytosis rhythm was advanced in spring; however, the peaks phagocytic activity occurred during light phase in both seasons as sunset occurs at around 19.00 h in summer in Varanasi.

It has been demonstrated that macrophages possess autonomous molecular clock machinery, which is similar to those in suprachiasmatic nucleus (SCN) and the presence of molecular clock system in macrophages suggests that the functions of macrophages vary rhythmically. There are number of reports on rhythms in phagocytosis. Circadian time structure of the neutrophil phagocytic index has been described in human [26], rodents [7,9,27–30] and guinea pigs [31]. Phagocytic activity in peritoneal macrophages of mice peaked in the light period and bottomed in the dark period [32]. The peak of phagocytic activity has been reported during the light phase, as the maximum engulfment of carbon particles by reticuloendothelial cells in CBA mice occurs during the second half of the light span [27]; while phagocytes collected from different tissues of C57BL/6 mice have shown peak phagocytic activity in the first half of the light span [32,33]. Roy et al. [34] have also reported diurnal rhythm in phagocytic activity—being considerably higher during light phase than dark phase—of splenic phagocytes in fish. This is the first report on rhythms in phagocytic activity in an ophidian system.

In contrast, there are reports, wherein phagocytic activity has been shown to remain elevated during the dark phase. Polymorphonuclear cells exhibited diurnal periodicity with peak phagocytosis at midnight in human [26]. The heterophils in ring doves [35], neutrophils in rat [36], and mice [37], the peak of phagocytosis have been observed toward the end of the second half of dark span; whereas maximal phagocytic activity in guinea pig has been demonstrated during the first half of the dark phase [31]. However, the timings of the peak in phagocytic activity vary in different species of animals. On the other hand, Bongrand et al. [38] failed to observe any circadian rhythm in neutrophil phagocytic activity in human subjects. Comparison of different laboratory procedures suggests that differences in the peak timings could be attributed to the tested phase of phagocytosis and the sensitivity of the laboratory procedures [9]. It has been shown that phagocytic activity, the phagocytic index of small particles, and the absolute count of phagocytic cells all register the peaks at the end of the subjective day.

NBT reduction and nitrite release by splenic macrophages of fresh water snake also varied significantly along a 24-h time scale during winter season. The peaks in both the variables occurred in the morning hours at 10.88 h and 8.31 h, respectively. But, significant 24-h rhythm was absent in NBT reduction and nitrite release during summer and winter seasons (Tables 1, 3). The results of one-way ANOVA revealed statistically significant time effect for NBT reduction in both summer and spring and for nitrite release in summer only. The observed discrepancy between the results of the Cosinor analysis and the single factor ANOVA could be ascribed to possible presence of ultradian rhythms in NBT reduction and Nitrite release. A distinct bimodality in NBT reduction and Nitrite release during summer and spring was observed in the present study (Fig. 1). The present results do not appear to be unusual as various components of the mammalian immune system have been known to be characterized by a multi-frequency time structure [39–42]. Therefore, it would be worthwhile to design studies to investigate the characteristics of multi-frequency rhythms in the immune system of ophids and other reptiles.

Circadian rhythm in superoxide anion production by inflammatory neutrophil has also been demonstrated in mice [43] and rat [9]. Similar daily rhythms in chemotaxis and neutrophil migration have been described in human, mice and cows [7–9]. Diurnal rhythms in innate immune functions have been described in fishes, gilthead seabream and sea bass [44]. Both, the time of exposure of immunocompetent cells to an antigen [45,46] and the response to challenge of the immunized organism after the introduction of the antigen [45–49] show circadian variations in human subjects and animals. Circadian rhythm in the natural killer cells, in T and B cells has also been described in many animals [46,50].

A statistically significant 24-h rhythm was also observed in lysozyme concentration in all three seasons: summer, winter and spring. The peak lysozyme concentration was found around the midnight in all three seasons. The peaks of lysozyme concentration did not phase-shift significantly along the seasonal time scale (Fig. 3B), although the peaks in spring (23.41 h) and summer (22.13 h) was slightly earlier than that in winter (1.49 h) season.

Statistically significant 24-h rhythms were observed in basal and mitogen (Con A) induced splenocyte proliferation in all three seasons: summer, winter and spring (Tables 1, 2, 3). The peaks in basal splenocyte proliferation rhythm occurred significantly earlier in winter than spring and summer seasons. The peak in winter was in the early morning at 4.35 h. The peak of the same variable was significantly earlier in spring at late morning hour (11.47 h) than summer, when the peak appeared in the night at 21.41 h. The peaks of Con A stimulated splenocyte proliferation also demonstrated comparable trend like the Basal splenocyte proliferation. A significant phase shift in this variable was witnessed in all three seasons with a trend of delayed phase shift from winter to spring and from spring to summer. The peak of mitogen induced splenocyte proliferation in winter was in the early morning (5.08 h), in spring at 11.47 h and in summer the peak was noticed at night (21.32 h). However, in spring season, the peaks of the both basal and Con A10 stimulated splenocyte proliferation rhythms coincidentally appeared at the same time in the late morning hours (11.47 h).

Circadian rhythms have been reported in PHA-induced proliferative responses of circulating lymphocytes in human subjects [51] and splenic cells of mice [2]. The mammalian lymphatic system has been reported to show a circadian time structure with phase differences in the responsiveness and in the proliferation of its components. The response of splenic cells to the mitogen (PHA) studied *in vitro* in 72 h cultures of cells obtained at six time points over a 24-h span from mice, the uptake of ^3^H-thymidine in splenic cells *in vitro* was found to be circadian in nature, with and without the PHA in the culture medium. The circadian rhythm of spontaneous incorporation of ^3^H-thymidine in the DNA of immunocompetent mammalian cells suggests a circadian rhythm in their proliferative activity. The response of these cells to mitogen, measured *in vitro* 72 h after removal of the cells from the body, varied as a function of the circadian stage in which the cells were collected [2]. This suggests to the existence some mechanism that enables circadian oscillators to encode time memory into the immunocompetent cells. Such a possibility has been examined and demonstrated in golden hamster recently [52]. Since ophidian immune system is evolutionarily older to the mammalian immune system it would be interesting to look for time memory in the immunocompetent cells in ophids and other reptiles.

Notwithstanding the observed 24-h rhythms in various immune parameters of the snake, statistically significant annual rhythms were observed in seven out of nine tested variables (Table 4; Fig. 2). Significant seasonal rhythms were absent only in the phagocytic index and the splenosomatic index (Table 4). The annual (= seasonal) peak map indicates that NBT reduction, Nitrite release, phagocytic index, basal/ mitogen (Con A) induced splenocyte proliferation, and lysozyme concentration exhibited a spectacular internal synchronization; the spread of the peaks in all these six variables overlapped with each other and occurred mostly between July and August, with the exception of spleen cellularity that exhibited a peak in November. The significant seasonal rhythms were validated by the Cosinor rhythmometry and complemented by the results of 2-way ANOVA (Table 5). The values of R^2^ (Table 5) strongly suggest that a higher percentage of data of all tested variables, except the indices, contributed significantly to the observed seasonality. There are very few papers on seasonality in immune system of reptiles [53–56], and the least is known in snakes; therefore, it is difficult to have a comparative account of seasonality of immune system in snakes. The tightly synchronized seasonal rhythms observed in the immune parameters with a narrow peak spread in July-August could be attributed to the progressive and beginning of the reproductive phase in *Natrix piscator*. The reproductive cycle of this snake has been reported elsewhere [57]. Probably this phase is important for the species to ensure that it counteracts all antigenic invasions effectively in order to have a successful reproductive outcome.

In summary, it is evident from the present study that the snake, *Natrix piscator* possesses a well-coordinated temporal mechanism in its immune system. It synchronizes its immune activity according to the time of the day/ season and probably vis-à-vis rhythmicity in prevalence of pathogens and manifestation of diseases. *Natrix piscator* is a seasonal breeder and the observed seasonality in its immune function probably provides a congenial immune status for the preparation and initiation of its annual breeding activity. Perhaps this adaptation is provided by positive ecological factors (temperature, photoperiod and humidity) and ample food availability during the reproductively active phase. Enhancement of immune function and its timing might be helping this seasonal breeder to cope with seasonal stressors that would otherwise compromise accomplishment of successful reproduction.

## References

[1] Scheving EL, Pauly JE, Tsai T (1968) Circadian fluctuation in plasma protein of the rat. Am J Physiol 215: 1097–1101. 10.1152/ajplegacy.1968.215.5.1096 4176820

[2] Haus E, Lakatua D, Swoyer J, Sackett-Lundeen L (1983) Chronobiology in hematology and immunology. Am J Anat 168: 467–517. 6364772 10.1002/aja.1001680406

[3] Mauer AM (1965) Diurnal variations of proliferative activity in the human bone marrow. Blood 26: 1–7. 14314397

[4] Clark RH, Korst DR (1969) Circadian periodicity of bone marrow mitotic activity and reticulocyte counts in rats and mice. Science 166: 236–237. 5809594 10.1126/science.166.3902.236

[5] Aardal NP, Laerum OD (1983) Circadian variations in mouse bone marrow. Exp Hematol 11: 792–801. 6641825

[6] Levi F, Canona C, Depres-Brummera P, Adam R, Bourina P, et al (1992) The rhythmic organization of the immune network: implications for the chronopharmacologic delivery of interferons, interleukins and cyclosporine. Ad Drug Deliver Rev 9(1): 85–112.

[7] Bureau JP, Labrecque G (1996) Rhythms biogiques, inflammation et anti-inflammatories non steroidiens. Pathol Biol 44: 610–617. 8977918

[8] van Werven T, Vander-Broek J, Noordhuizen-Stassen EN (1996) Within day and between day variation of the *in vitro* under agarose chemotaxis assay in bovine. Vet Immunopathol. 55: 83–91. 9014308 10.1016/s0165-2427(96)05723-6

[9] Berger J, Slapnickova M (2003) Circadian structure of rat neutrophil phagocytosis. Comp. Clin Pathol 12: 84–89.

[10] Singh T, Singh R (2012) Circadian variation in peripheral blood leucocytes, the primary immune cells, in the garden lizard, *Calotis versicolor (Daudin)* . The Bioscan 7(2): 211–214.

[11] Pati AK, Thapliyal JP (1984) Circannual variation in the timing of eosinophil circadian rhythm in snake In: Chronobiology. 1982–83, (Eds Haus E, Kabat HF Basel: S Karger) pp 25–30.

[12] Pati AK (1989) Circannual modulation of circadian mesor of circulating eosinophil in an ophid. J Ravishankar Univ 2B: 18–22.

[13] Pati AK, Pradhan RK, Pathak VK, Saini SK, Biswas J (1987) Circadian rhythms in non mammalian vertebrates. Biome 2: 58–69.

[14] Mondal S, Rai U (1999a) Sexual dimorphism in phagocytic activity of wall lizard’s splenic macrophages and its control by sex steroids. Gen Com Endocrinol 116: 291–298. 10562459 10.1006/gcen.1999.7370

[15] Mondal S, Rai U (1999b) Dose-dependent effect of sex-steroids in lizard’s splenic macrophage phagocytic activity 3rd International Symposia of Asia and Oceania society for Comparative Endocrinology, Republic of Korea pp. 482–488.

[16] Keller JM, McClellan-Green PD, Lee AM, Arendt MD, Maire PP, et al (2005) Mitogen-induced lymphocyte proliferation in loggerhead sea turtles: comparison of methods and effects of gender, plasma testosterone concentration, and body condition on immunity. Vet Immunol Immunopathol 103: 269–281. 15621312 10.1016/j.vetimm.2004.09.029

[17] Jorens PG, Matthys KE, Bult H (1995) Modulation of nitric oxide synthase activity in macrophages. Mediators Inflamm 4: 75–89. 18475620 10.1155/S0962935195000135 PMC2365621

[18] Ding AH, Nathan CF, Stuehr DJ (1988) Release of reactive nitrogen intermediated and reactive oxygen intermediates from mouse peritoneal macrophages: comparison of activating cytokines and evidence for independent production. J Immunol 141: 2407–2412. 3139757

[19] Berridge MV, Herst PM, Tan AS (2005) Tetrazolium dyes as tools in cell biology: New insights into their cellular reduction. Biotechnol Annu Rev 11: 127–152. 16216776 10.1016/S1387-2656(05)11004-7

[20] Mosmann T (1983) Rapid colorimetric assay for cellular growth and survival: application to proliferation and cytotoxicity assays. J Immunol Methods 65: 55–63. 6606682 10.1016/0022-1759(83)90303-4

[21] Cory AH, Owen TC, Barltrop JA, Cory JG (1991) Use of an aqueous soluble tetrazolium/formazan assay for cell growth assays in culture. Cancer Commun 3: 207–212. 1867954 10.3727/095535491820873191

[22] Gieni RS, Li Y, Hay, Glass KT (1995) Comparison of [^3^H]thymidine incorporation with MTT- and MTS-based bioassays for human and murine IL-2 and IL-4 analysis. Tetrazolium assays provide markedly enhanced sensitivity. J Immunol Methods 187: 85–93. 7490461 10.1016/0022-1759(95)00170-f

[23] Demers NE, Bayne CJ (1997) The immediate effects of stress on hormones and plasma lysozyme in rainbow trout. Dev Comp Immunol 21(4): 363–373. 9303274 10.1016/s0145-305x(97)00009-8

[24] Gupta S, Pati AK (1992) Data analysis methodology in chronobiological studies. J Parasitol Appl Anim Biol 1:151–163.

[25] Nelson W, Tong YL, Lee JK, Halberg F (1979) Method for cosinor rhythmometry. Chronobiologia 6:305–323. 548245

[26] Melchart D, Martin P, Hallek M (1992) Circadian variation of phagocytic activity of polymorphonuclear leukocytes and of various other parameters in 13 healthy male adults. Chronobiol Int 9: 35–45. 1555260 10.3109/07420529209064514

[27] Szabo I, Kovat STG, Halberg F (1978) Circadian rhythm in murine reticuloendothelial function. Chronobiologia 5: 137–143. 679796

[28] Berger J (2004) Chronohematology. J App Biomed 2: 179–185.

[29] Hriscu ML (2004) Circadian phagocytic activity of neutrophils and its modulation by light. J Appl Biomed 2: 199–211.

[30] Hriscu ML (2005) Modulatory factors of circadian phagocytic activity. Ann NY Acad Sci 1057: 403–430. 16399910 10.1196/annals.1356.032

[31] Baciu I, Olteanu A, Prodan T (1988) Changes of phagocytic biological rhythm by reduction of circadian times and by influences upon hypothalamus. Int J Neurosci 41: 143–153. 3137183 10.3109/00207458808985750

[32] Hayashi M, Shimba S, Tezuka M (2007) Characterization of the molecular clock in mouse peritoneal macrophages. Biol Pharm Bull 30: 621–626. 17409491 10.1248/bpb.30.621

[33] Knyszynski A, Fischer H (1981) Circadian fluctuations in the activity of phagocytic cells in blood, spleen and peritoneal cavity of mice as measured by zymozan induced chemiluminescence. J Immunol 127: 2508–2511. 7299134

[34] Roy B, Singh R, Kumar S, Rai U (2008) Diurnal variation in phagocytic activity of splenic phagocytes in freshwater teleost *Channa punctatus*: melatonin and its signalling mechanism. J Endocrinol 199: 471–480. doi: 10.1677/JOE-08-0270 18824520 10.1677/JOE-08-0270

[35] Rodriguez AB, Marchena JM, Nogales G, Duram J, Barriga C (1999) Correlation between the circadian rhythm of melatonin, phagocytosis, and superoxide anion levels in ring dove heterophils. J Pineal Res 26: 35–42. 10102758 10.1111/j.1600-079x.1999.tb00564.x

[36] Hriscu M, Saulea G, Ostriceanu S, Baciu I (2002–2003) Circadian phagocytic activity in rats under light—dark and constant light regimens. Rom J Physiol 40: 17–26. 15984664

[37] Hriscu M, Saulea G, Vidrascu N, Baciu I (1998) Circadian rhythm of phagocytosis in mice. Rom J Physiol 35: 319–323. 11061331

[38] Bongrand P, Bouvenot G, Bartolin R (1988) Are there circadian variations of polymorphonuclear phagocytosis in man? Chronobiol Int 5: 81–83. 3370719 10.3109/07420528809078554

[39] Levi F, Canon C, Blum JP, Mechkouri M, Reinberg A, et al (1985) Circadian and/or circahemidian rhythms in nine lymphocyte-related variables from peripheral blood of healthy subjects. J Immunol 134: 217–222. 3855259

[40] Pati AK, Florentin I, Chung V, De Sousa M, Levi F, et al (1987) Circannual rhythm in natural killer activity and mitogen responsiveness of murine splenocytes. Cell immunol 108 (1): 227–234. 3496974 10.1016/0008-8749(87)90207-3

[41] Fernandes G (1992) Chronobiology of immune functions: Cellular and humoral aspects Pp. 493–503 in Biologic Rhythms in Clinical and Laboratory Medicine, Touitou Y., editor; and Haus E., editor., eds. Heidelberg: Springer-Verlag.

[42] Haus E, Smolensky MH (1999) Biologic Rhythms in the Immune System. Chronobiol Int 16(5): 581–622. 10513884 10.3109/07420529908998730

[43] Brigagao MRPL, Colepicolo P (1998) Activation of neutrophils is daily inhibited by saliva. Biol Rhythm Res 29: 598–605.

[44] Esteban MA, Cuesta A, Rodrıguez A, Meseguer J (2006) Effect of photoperiod on the fish innate immune system: a link between fish pineal gland and the immune system. J Pineal Res 41: 261–266. 16948787 10.1111/j.1600-079X.2006.00362.x

[45] Fernandes G, Yunis EJ, Halberg F (1977) Circadian aspect of immune responses in the mouse In Chronobiology in Allergy and Immunology (McGovern JP, Smolensky MH, Reinberg A, Eds) pp. 233–249. Thomas, Springfield, IL.

[46] Fernandes G, Halberg G, Good APA (1980) Circadian rhythm in T, B and natural killer cells Recent Advances in the Chronobiology of Allergy and Immunology. (Smolensky MH Ed.) pp. 289–299. Pergamon Oxford/New York.

[47] Fernandes G, Halberg F, Yunis EJ, Good RA (1976) Circadian Rhythmic PlaqueForming Cell Response of Spleens from Mice Immunized with SRBC. J Immunol 117: 962–966. 986413

[48] Lee P, Murray BU, Arthur AMB, Barry EK, Hugh AS, et al (1977) Systemic Lupus Erythematosus: A review of 110 cases with reference to nephritis, the nervous system, infections, aseptic necrosis and prognosis. Q J Med 46(1): 1–32. 866565

[49] Cove-Smith JR, Pownall R, Kabler TA, Knapp MS (1979) Circadian variation in cell-mediated immune response in man and their response to prednisolone In: Reinberg A, Halberg F (eds.) Chronopharmacol Pergamon Oxford pp. 369–374.

[50] Abo T, Kawate T, Itoh K, Kumagai K (1981) Studies on the bioperiodicity of the immune response, I. Circadian rhythms of human T, B, and K cell traffic in the peripheral blood. J Immunol 126: 1360–1363. 6970770

[51] Eskola J, Frey H, Molnar G, Sppi E (1976) Biological rhythm of cell mediated immunity in man. Clin Exp Immunol 26: 253–257. 1033049 PMC1540849

[52] Ralph MR, Sam K, Rawashdeh OA, Cain SW, Ko CH (2013) Memory for time of day (time memory) is encoded by a circadian oscillator and is distinct from other context memories. Chronobiol Int 30(4):540–547. doi: 10.3109/07420528.2012.754449 23428333 10.3109/07420528.2012.754449

[53] Hussein MF, Badir N, El Ridi R, Akef M (1978) Differential effect of seasonal variation on lymphoid tissue of the lizard, *Chalcides ocellatus* . Dev Comp Immunol. 2, 297–310. 680304 10.1016/s0145-305x(78)80072-x

[54] Munoz FJ, Galvan A, Lerma M, De la Fuente M (2000) Seasonal changes in peripheral blood leukocyte functions of the turtle *Mauremys caspica* and their relationship with corticosterone, 17 beta-estradiol and testosterone serum levels. Vet Immunol Immunopathol. 77: 27–42. 11068064 10.1016/s0165-2427(00)00228-2

[55] Munoz FJ, De la Fuente M (2001) The immune response of thymic cells from the turtle *Mauremys caspica* . J Comp Physiol B. 171(3):195–200. 11352102 10.1007/s003600000159

[56] Sherif M, El Ridia R (1992) Natural Cytotoxic Cell Activity in the Snake *Psammophis sibilans* . Immunobiol. 184(4–5):348–358. 10.1016/S0171-2985(11)80592-9 1592427

[57] Haldar C, Pandey R (1989) Effect of pinealectomy on annual testicular cycle of Indian chequered water snake, *Natrix piscator* . Gen Comp Endocrinol. 76(2): 214–222. 2591715 10.1016/0016-6480(89)90152-4

